# Change in the Editorial Leadership of *PLoS Pathogens*

**DOI:** 10.1371/journal.ppat.0040015

**Published:** 2008-01-25

**Authors:** Kasturi Haldar, Grant McFadden, John A. T Young

Dear Readers,

In the fall of 2004, John Young was approached by Harold Varmus and the PLoS founders to start a new PLoS journal in the field of microbial pathogenesis. The need for such a journal was immediately recognized by the community. Since the initial call for papers in 2005, the journal has grown steadily and, at times, dramatically, with consistent increases in both the number of manuscripts considered and manuscripts published, and with a significant expansion of the Editorial Board. Under John's direction, and by dint of the tireless and outstanding work of the Editorial Board, each of whom volunteer their time, *PLoS Pathogens* has lived up to its stated goals of publishing “the highest quality work that embodies a significant advance across microbial pathogenesis.”

John's leadership is manifest at all levels of the journal, and we now encounter our biggest editorial transition to date as he steps aside as Editor-in-Chief. Particularly important to this transition has been the development of a journal editorial structure that ensures a smooth adjustment of responsibilities as board members balance the complex demands of time, both professional and personal. In this way, former Deputy Editor Kasturi Haldar will now assume EIC duties, and Grant McFadden, one of the Virology Section Editors, will take on the Deputy Editor role; John will remain an active member of the community and, in the role of Associate Editor, the journal.

We are indebted to John for his expert guidance of the journal and expect to continue to build off of his example. We are also naturally excited about the possibilities inherent with changes in journal leadership, pathogens research, and open-access publishing. We look forward to another dynamic year ahead with you, the community of *PLoS Pathogens*.

Thanks for your continued support and best wishes,

Kasturi, Grant, and John

